# Involvement of Nitric Oxide and Melatonin Enhances Cadmium Resistance of Tomato Seedlings through Regulation of the Ascorbate–Glutathione Cycle and ROS Metabolism

**DOI:** 10.3390/ijms24119526

**Published:** 2023-05-31

**Authors:** Junrong Xu, Zhien Wei, Xuefang Lu, Yunzhi Liu, Wenjin Yu, Changxia Li

**Affiliations:** College of Agriculture, Guangxi University, Nanning 530004, China

**Keywords:** nitric oxide, melatonin, Cd stress, AsA-GSH cycle, regulator pathway, gene expression

## Abstract

Melatonin (MT) and nitric oxide (NO) act as signaling molecules that can enhance cadmium (Cd) stress resistance in plants. However, little information is available about the relationship between MT and NO during seedling growth under Cd stress. We hypothesize that NO may be involved in how MT responds to Cd stress during seedling growth. The aim of this study is to evaluate the relationship and mechanism of response. The results indicate that different concentrations of Cd inhibit the growth of tomato seedlings. Exogenous MT or NO promotes seedling growth under Cd stress, with a maximal biological response at 100 μM MT or NO. The promotive effects of MT-induced seedling growth under Cd stress are suppressed by NO scavenger 2-4-carboxyphenyl-4,4,5,5-tetramethyl-imidazoline-1-oxyl-3-oxide (cPTIO), suggesting that NO may be involved in MT-induced seedling growth under Cd stress. MT or NO decreases the content of hydrogen peroxide (H_2_O_2_), malonaldehyde (MDA), dehydroascorbic acid (DHA), and oxidized glutathione (GSSG); improves the content of ascorbic acid (AsA) and glutathione (GSH) and the ratios of AsA/DHA and GSH/GSSG; and enhances the activities of glutathione reductase (GR), monodehydroascorbic acid reductase (MDHAR), dehydroascorbic acid reductase (DHAR), ascorbic acid oxidase (AAO), and ascorbate peroxidase (APX) to alleviate oxidative damage. Moreover, the expression of genes associated with the ascorbate–glutathione (AsA-GSH) cycle and reactive oxygen species (ROS) are up-regulated by MT or NO under Cd conditions, including *AAO*, *AAOH*, *APX1*, *APX6*, *DHAR1*, *DHAR2*, *MDHAR*, and *GR*. However, NO scavenger cPTIO reverses the positive effects regulated by MT. The results indicate that MT-mediated NO enhances Cd tolerance by regulating AsA-GSH cycle and ROS metabolism.

## 1. Introduction

Cadmium (Cd) is a common heavy metal [1] and is considered to be one of the most toxic heavy metals [2]. In the soil, Cd is added constantly by smelting and mining, rock weathering, and the overuse of phosphate fertilizers [3,4,5]. Cd is a non-essential element for plant growth [6] and some Cd compounds are absorbed by roots in the soil and transported throughout the plant resulting in Cd accumulation [7,8]. Because Cd has strong affinity for sulfhydryl moiety of enzymes, it can affect plant metabolism by inhibiting enzyme activities [9]. Meanwhile, Cd absorption can disrupt nutrient uptake [10] and homeostasis [11] causing negative effects on physiology and morphology such as seed germination [12], seedling growth [12], chlorophyll degradation [13], photosynthesis inhibition [14], nitrogen metabolism [15], and plant biomass [16]. Interestingly, when Cd content exceeds a certain amount in plants, reactive oxygen species (ROS) formation is enhanced [15], including hydroxyl radicals (OH^−^), superoxide radicals (O_2_^−^), and hydrogen peroxide (H_2_O_2_) [17]. ROS is a double-edged sword: low concentration is able to increase plant tolerance, but high concentration can lead to plasma membrane oxidization of plant cells [18]. In order to eliminate excess ROS, plants have developed a variety of physiological and biochemical mechanisms [19], for example, antioxidants and antioxidant enzymes [20]. Previous studies have shown that there are three main Cd resistance strategies in plants: the first strategy is absorption of Cd or isolation of Cd inside the plant; the second is alleviation of Cd toxicity and Cd removal through a series of chelating mechanisms; and the third is removal of ROS that accumulate during Cd stress [21,22].

Nitric oxide (NO) acts as a signaling molecule that is involved in a wide variety of developmental processes [23,24]. Furthermore, NO plays an important role in alleviation of abiotic stresses. For example, NO mediates cucumber adventitious rooting through inducing the production of methane (CH_4_) [25]. Hydrogen (H_2_)-mediated NO regulates the expression levels and interactions of plasma membrane H^+^-ATPase and 14-3-3 protein to induce adventitious rooting [26]. More importantly, NO can crosstalk with other plant hormones to regulate a series of physiological processes. For instance, Cao et al. [27] demonstrate that auxin-induced H_2_ production is associated with lateral root formation, at least partially via a nitrate reductase (NR)-dependent NO synthesis.

Melatonin (MT) is known as a pleiotropic molecule in higher plants due to its broad and diverse physiological functions. A growing body of research suggests that MT has multiple roles in plant growth and development [28,29]. In addition, MT is considered an efficient antioxidant that can be used to protect plants from abiotic stresses and enhance abiotic tolerance, including drought [30], salt stress [31], cold stress [32,33], light stress [34], and heavy metal stress [35]. Recently, it has been shown that one of these ROS-regulated molecules is MT. Interestingly, ROS is able to up-regulate the MT biosynthesis pathway genes, thereby increasing the plant’s endogenous levels of MT [36]. In turn, endogenous MT can act as an effective ROS scavenger [37,38]. Thus, MT controls ROS levels in two different ways: through its chemical interaction with ROS which leads to ROS inactivation [29]; or by MT-mediated induction of the redox enzymes that detoxify ROS, such as superoxide dismutase (SOD), catalase (CAT), peroxidase (POD), glutathione peroxidase (GPX), and ascorbate peroxidase (APX) [39]. On the other hand, MT also induces the accumulation of some representative non-enzymatic antioxidant compounds such as glutathione (GSH), ascorbic acid (AsA) [40,41,42,43,44,45,46,47], phenolic compounds [48], flavonoid via the NO-dependent pathway [49], and carotenoids [50]. Interestingly, researchers have reported that crosstalk between MT and NO can regulate some physiological processes. For instance, Liu et al. [51] show that MT depends on NO to respond to low-temperature stress in litchi fruit. MT delays postharvest senescence by inhibiting ethylene biosynthesis and this process requires the involvement of NO [52]. MT can induce NO production or scavenge excess NO and can promote the accumulation of NO by increasing the activity of NO synthase (NOS)-like protein (arginine metabolic pathway), as MT up-regulates the expression of related genes [33,52]. The interaction between NO and MT shows a certain degree of intricacy, as they interact independently and through multiple signaling pathways [53].

As mentioned above, in various model plants or crop species, the roles of NO or MT in regulating the process of plant growth, development, and responses to stress stimuli have been extensively reported. However, the regulatory mechanisms of MT and NO under Cd stress during seedling growth remain unclear. The objective of this study is to explore the mechanisms of interaction between MT and NO during seedling growth under Cd stress. Therefore, these findings imply a relationship between MT and NO in responding to abiotic stress in plants.

## 2. Results

### 2.1. Effect of Different Concentrations of Cd on the Growth of Tomato Seedlings

**Figure 1:**
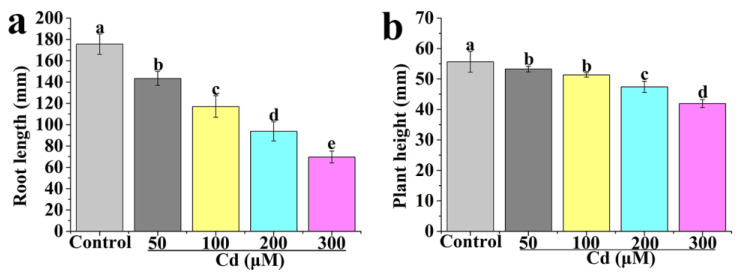
Effects of different concentrations of CdCl_2_ on root length (**a**) and plant height (**b**) of tomato seedlings after 7 days. Values are presented as mean ± SE calculated from at least three independent experiments (15 seedlings per replication). Bars denoted by the same letter indicate no significant difference at *p* < 0.05 according to Tukey’s test.

The root length and plant height of tomato seedlings treated with different concentrations of Cd are measured (Figure 1). The overall trends of root length and plant height decline with the increase of Cd concentration and Cd exhibits a dose-dependent effect on root length and plant height of tomato seedlings. Compared with the control, treatments with 50, 100, 200, and 300 μM Cd result in a significant decrease in root length, from 175.64 to 143.39, 117.09, 93.72, and 69.73, respectively (Figure 1a). The change of plant height (Figure 1b) is similar. These results indicate that treatments with 50–100, 200, and 300 μM Cd can be termed as mild, moderate, and severe Cd stress, respectively. Therefore, 200 μM Cd (moderate stress) is selected for further studies. Meanwhile, the selection of 200 μM Cd is similar to previous studies [54,55].

### 2.2. Effect of Different Concentrations of MT and SNP on the Growth of Tomato Seedlings under Cd Stress

**Figure 2:**
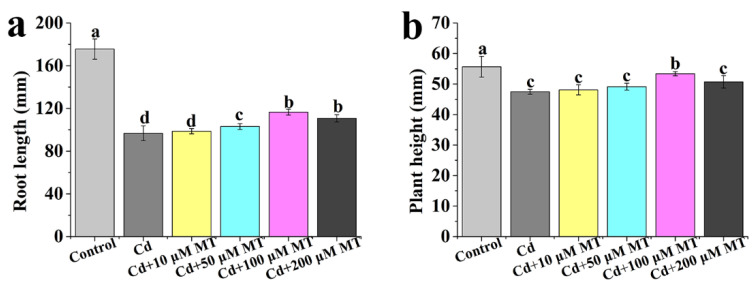
Effects of different concentrations of MT on root length (**a**) and plant height (**b**) of tomato seedlings under Cd stress. Seedlings are incubated in Hoagland solution containing 200 μM CdCl_2_ added at different concentrations of MT. Values are presented as mean ± SE calculated from at least three independent experiments (15 seedlings per replication). Bars denoted by the same letter indicate no significant difference at *p* < 0.05 according to Tukey’s test.

The root length is remarkably reduced by Cd compared to control (Figure 2a). There is no significant change in root length between Cd and Cd + 10 μM MT (Figure 2a). Compared to Cd + 10 μM MT, the inhibition of root length caused by Cd is significantly increased by 50, 100, or 200 μM MT, while there is no significant change in root length between Cd + 100 μM MT and Cd + 200 μM MT (Figure 2a), suggesting that the maximum alleviation in root length is obtained at 100 μM MT under Cd stress. Similarly, the Cd-caused decrease in plant height is alleviated by 100 μM MT (Figure 2b). Combined with the previous results shown in Figure 2, these results suggest that MT (100 μM) is the most effective under our experimental conditions. Therefore, 100 μM MT is used for further studies.

**Figure 3:**
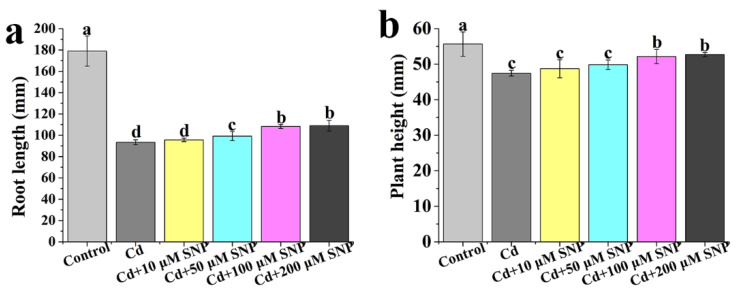
Effects of different concentrations of SNP on root length (**a**) and plant height (**b**) of tomato seedlings under Cd stress. Values are presented as mean ± SE, calculated from at least three independent experiments (15 seedlings per replication). Bars denoted by the same letter indicate no significant difference at *p* < 0.05 according to Tukey’s test.

Cd stress results in a significant decrease in the root length of seedlings, but 50, 100, or 200 μM SNP all significantly alleviate the decrease (Figure 3a). There is no significant change in root length between 100 μM SNP and 200 μM SNP (Figure 3a). Thus, 100 μM SNP as the most effective is able to alleviate the inhibition of root length. Under Cd stress, the effect of different concentrations of SNP on plant height is similar to the change in root length (Figure 3b). Therefore, 100 μM SNP is the most effective for alleviating Cd stress and is used for further studies.

### 2.3. Effect of Different Treatments on the Growth of Tomato Seedlings under Cd Stress

**Figure 4:**
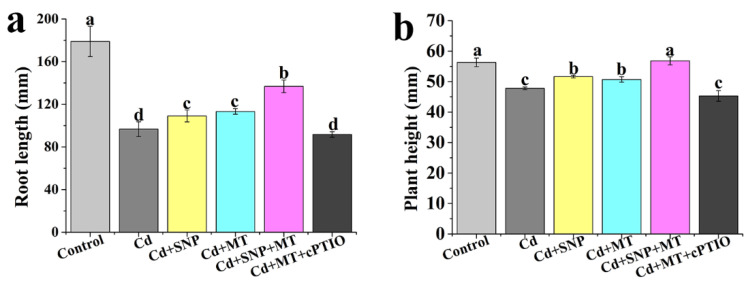
Effect of MT, SNP, MT + SNP, and MT + cPTIO on root length (**a**) and plant height (**b**) of tomato seedlings under Cd stress. Values are presented as mean ± SE calculated from at least three independent experiments (15 seedlings per replication). Bars denoted by the same letter indicate no significant difference at *p* < 0.05 according to Tukey’s test.

To study the relationship between MT and NO in the promotion of tomato seedlings under Cd stress, SNP, MT, and NO scavenger cPTIO are used in the study. Compared with the control, seedlings treated with Cd alone exhibited a significant decrease in the root length and plant height (Figure 4a,b). SNP or MT treatment significantly increases the root length and plant height under Cd stress, and SNP and MT co-treatment significantly enhances the root length and plant height in comparison with SNP or MT alone under Cd stress (Figure 4a,b). Seedlings treated with Cd + MT exhibited a decrease in the root length and plant height with the addition of cPTIO (Figure 4a,b). Thus, NO may be involved in MT-induced Cd stress tolerance.

### 2.4. Different Treatments Alter the Levels of H_2_O_2_ and MDA under Cd Stress

**Figure 5:**
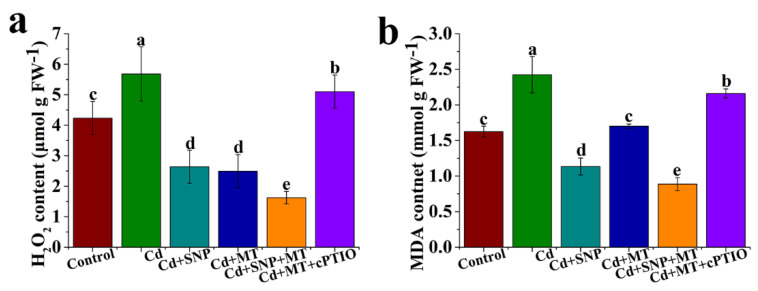
The levels of H_2_O_2_ (**a**) and MDA (**b**) under MT, SNP, MT + SNP, and MT + cPTIO treatments after 7 days. Values are presented as mean ± SE calculated from at least three independent experiments (15 seedlings per replication). Bars denoted by the same letter indicate no significant difference at *p* < 0.05 according to Tukey’s test.

As shown in Figure 5, the levels of H_2_O_2_ and MDA are elevated under Cd treatment compared to the control. Seedlings treated with SNP or MT alone exhibited a significant decrease in the H_2_O_2_ and MDA contents under Cd stress (Figure 5). Meanwhile, H_2_O_2_ and MDA levels are obviously reduced by SNP + MT treatment under Cd stress compared to SNP or MT treatment alone (Figure 5). When cPTIO is applied, MT-reduced H_2_O_2_ and MDA levels are reversed under Cd stress (Figure 5).

### 2.5. Effect of Different Treatments on the Levels of AsA, DHA, GSH, and GSSG under Cd Stress

**Figure 6:**
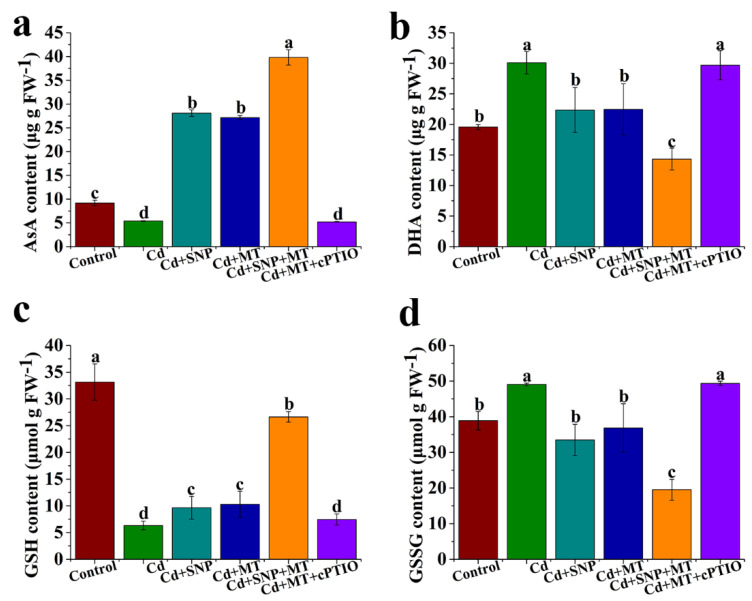
The levels of AsA (**a**), DHA (**b**), GSH (**c**), and GSSG (**d**) under MT, SNP, MT + SNP, and MT + cPTIO treatments after 7 days. Values are presented as mean ± SE calculated from at least three independent experiments (15 seedlings per replication). Bars denoted by the same letter indicate no significant difference at *p* < 0.05 according to Tukey’s test.

Cd stress significantly reduces AsA content, but AsA level is increased by SNP or MT treatment alone (Figure 6a). Meanwhile, AsA content is promoted significantly by SNP + MT compared to SNP or MT treatment alone (Figure 6a). Moreover, AsA content is significantly decreased when cPTIO is added to MT treatment under Cd stress (Figure 6a). DHA content is increased by Cd stress. Compared with Cd treatment, the DHA content of treatment with Cd + SNP or Cd + MT decreases remarkably. The DHA content of treatment with Cd + SNP + MT is less than that of Cd + SNP or Cd + MT treatment. The decrease of DHA content caused by Cd + MT is distinctly increased by Cd + MT + cPTIO (Figure 6b). The change of GSH is similar to AsA content (Figure 6c). Likewise, the change of GSSG is similar to DHA content (Figure 6d).

### 2.6. Effect of Different Treatments on the Levels of AsA/DHA and GSH/GSSG Ratios under Cd Stress

**Figure 7:**
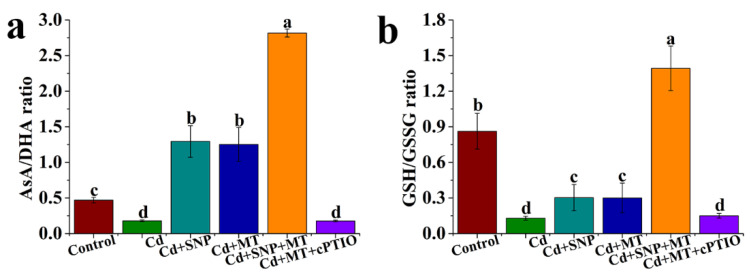
The levels of AsA/DHA (**a**) and GSH/GSSG (**b**) ratios MT, SNP, MT + SNP, and MT + cPTIO treatments after 7 days under Cd stress conditions. Values are presented as mean ± SE, which are calculated from at least three independent experiments (15 seedlings per replication). Bars denoted by the same letter indicate no significant difference at *p* < 0.05 according to Tukey’s test.

Compared with the control, Cd treatment decreases distinctly with AsA/DHA ratio, but this effect is improved by SNP or MT treatment (Figure 7a). The AsA/DHA ratio of treatment with Cd + SNP + MT is more than that of Cd + SNP or Cd + MT treatment (Figure 7a). Meanwhile, the MT-induced increase of the AsA/DHA ratio is inhibited by cPTIO under Cd stress conditions (Figure 7a). Cd stress significantly reduces GSH/GSSG ratio, but the inhibition is improved by SNP or MT (Figure 7b). Compared to SNP or MT treatment alone, SNP + MT significantly enhances GSH/GSSG ratio under Cd stress (Figure 7b). The GSH/GSSG ratio of treatment with Cd + MT is more than that of Cd + MT + cPTIO treatment (Figure 7b).

### 2.7. Effect of Different Treatments on the AAO and APX Activities and Gene Expression under Cd Stress

**Figure 8:**
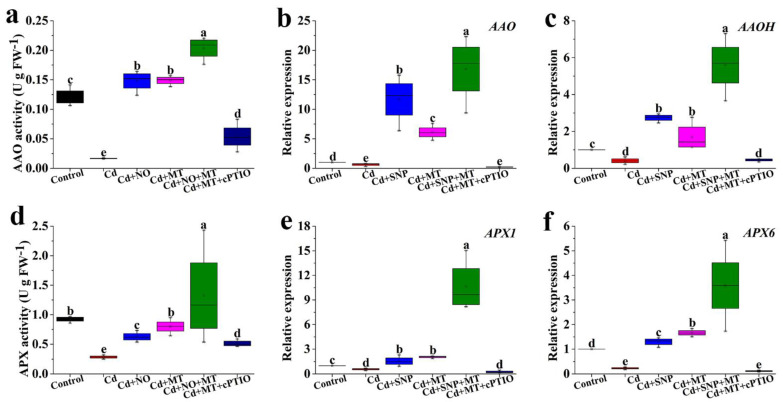
The activities of AAO (**a**) and APX (**d**) and the relative expression levels of *AAO* (**b**), *AAOH* (**c**), *APX1* (**e**), and *APX6* (**f**) genes under MT, SNP, MT + SNP, and MT + cPTIO treatments after 7 days. Values are presented as mean ± SE calculated from at least three independent experiments (15 seedlings per replication). Bars denoted by the same letter indicate no significant difference at *p* < 0.05 according to Tukey’s test.

Compared to the control, the activity of AAO is significantly reduced by Cd stress (Figure 8a). Nevertheless, AAO activity is significantly enhanced by SNP or MT treatment under Cd stress conditions, and AAO activity in the Cd + SNP + MT treatment is more than that of Cd + SNP or Cd + MT treatment (Figure 8a). When cPTIO is applied in MT treatment, its activity is significantly blocked compared to MT treatment under Cd stress (Figure 8a). The changes of *AAO* and *AAOH* genes are similar to AAO activity under different treatments (Figure 8b,c). The activity of APX treated with Cd is significantly lower than the control (Figure 8d). SNP or MT treatment significantly increases the APX activity under Cd stress, and SNP + MT co-treatment significantly increases the APX activity compared to SNP or MT treatment alone under Cd stress (Figure 8d). Cd + MT + cPTIO treatment has a significant inhibitory effect on APX activity compared with Cd + MT treatment (Figure 8d). The changes of *APX1* and *APX6* genes are similar to APX activity under different treatments (Figure 8e,f).

### 2.8. Effect of Different Treatments on the Activities of MDHAR, DHAR, and GR and Gene Expression under Cd Stress

**Figure 9:**
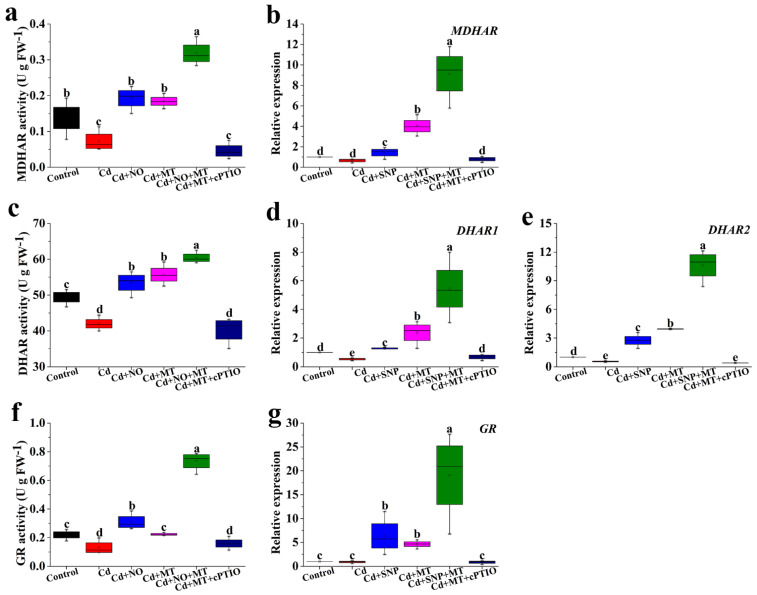
The activities of MDAHR (**a**), DHAR (**c**), and GR (**f**), and the relative expression levels of *MRHAR* (**b**), *DAHR1* (**d**), *DHAR2* (**e**), and *GR* (**g**) genes under MT, SNP, MT + SNP, and MT + cPTIO treatments after 7 days. Values are presented as mean ± SE calculated from at least three independent experiments (15 seedlings per replication). Bars denoted by the same letter indicate no significant difference at *p* < 0.05 according to Tukey’s test.

As shown in Figure 9a, Cd treatment significantly inhibits MDHAR activity compared with the control, whereas SNP or MT significantly reverses the inhibitive effect of Cd (Figure 9a). MDHAR activity of treatment with Cd + SNP + MT is higher than that of Cd + SNP or Cd + MT treatment (Figure 9a). Interestingly, Cd + MT + cPTIO treatment has a significant inhibitory effect on MDHAR activity compared with Cd + MT treatment (Figure 9a). These results are in full agreement with the change of the *MDHAR* gene (Figure 9b). SNP or MT significantly enhances the inhibition of DHAR activity caused by Cd stress, cPTIO significantly inhibits the increase enhanced by MT (Figure 9c). The changes of *DHAR1* and *DHAR2* genes are similar to DHAR activity (Figure 9d,e). Cd treatment significantly decreases GR activity compared with the control, whereas SNP or MT significantly reverses the inhibitive effect of Cd (Figure 9f). GR activity of treatment with Cd + SNP + MT is higher than that of Cd + SNP or Cd + MT treatment (Figure 9f). Interestingly, cPTIO significantly inhibits the increase of GR activity enhanced by MT (Figure 9f). These results are in full agreement with the change of the *GR* gene (Figure 9g).

## 3. Discussion

Cd is one of the most toxic heavy metal elements in nature and seriously influences plant growth and development [56]. Our data indicates that the root length and plant height of tomato seedlings are significantly decreased by Cd stress (Figure 1). MT and NO, as signaling molecules, are considered to alleviate abiotic stresses. For instance, Per et al. [57] point out that NO counteracts Cd toxicity in mustard. Exogenously applied MT can improve the ability of plants to tolerate Cd stresses [58]. In the study, MT or NO treatment significantly increases the root length and plant height of tomato seedlings under Cd stress (Figure 2 and Figure 3), indicating that MT or NO can alleviate Cd stress in tomato seedlings. However, the relationship between MT and NO in alleviating Cd stress is unclear. Under Cd stress, applied MT significantly increases the root length and plant height of tomato seedlings, whereas the effects are reversed with the addition of cPTIO (Figure 4), suggesting that NO may be involved in MT-induced improvement of Cd stress tolerance in tomato seedlings. Zhao et al. [59] indicate that NO operates downstream of MT in promoting salt tolerance. MT-mediated NO improves tolerance of lead (Pb) stress in maize [60]. MT can increase the iron (Fe) deficiency tolerance of plants dependent on polyamine-induced NO production under Fe-deficient conditions in *Arabidopsis* [61]. Thus, NO acts as a downstream signal in the MT-induced plant tolerance to abiotic stress.

Cd stress triggers the over-accumulation of ROS, ultimately resulting in oxidative stress in plants [62]. The levels of oxidative stress attributes, such as H_2_O_2_ and MDA, reflect the degree of oxidative stress. Our results indicate that MT or SNP treatment significantly reduces the increase in the H_2_O_2_ and MDA contents caused by Cd stress, but the additional application of cPTIO remarkably increases the reduced H_2_O_2_ and MDA contents by MT under Cd stress, suggesting that NO is involved in MT-mediated Cd stress by reducing the accumulations of H_2_O_2_ and MDA (Figure 5). MT significantly increases the NR activity, the transcript level of NR gene and endogenous NO content in cucumber seedlings under cold stress. Meanwhile, both MT and SNP decrease MDA and ROS accumulation by activating the antioxidant system and consequently reduce cold damage, implying that NO as a downstream signal of MT improves plant tolerance to cold stress [63]. Cd causes an increase in levels of H_2_O_2_ and MDA in wheat leaves, but MT reduces H_2_O_2_ and MDA content and increases endogenous NO production [64]. Simultaneously, MT-enhanced Cd stress tolerance is completely reversed by the supply of cPTIO, following which H_2_O_2_ and MDA contents are improved, thus suggesting that MT-mediated NO enhances Cd stress tolerance by reducing MDA and ROS accumulation [64]. Application of MT and NO significantly decreases ROS-induced lipid peroxidation under alkaline stress, but the effect of MT on ROS is greatly diminished by cPTIO, suggesting that MT responds to alkaline stress depending on the NO signaling pathway in tomato seedlings [65]. High levels of H_2_O_2_ and MDA are usually mediated by antioxidant enzymes, such as AAO and APX, which improve stress tolerance in plants. In the study, the Cd stress results in reduced AAO and APX activities, but AAO and APX activities are significantly enhanced by SNP or MT treatment alone (Figure 8). When cPTIO is applied in MT treatment, AAO and APX activities are significantly inhibited under Cd stress, following which the changes of *AAO*, *AAOH*, *APX1*, and *APX6* genes under different treatments are in full agreement with the AAO and APX activities (Figure 8), suggesting that NO is involved in MT-induced Cd stress tolerance by increasing AAO and APX activities, up-regulating the expression levels of *AAO*, *AAOH*, *APX1*, and *APX6* genes, thus resulting in reduced H_2_O_2_ and MDA. MT treatment increases APX activity during cold storage compared to control in litchi fruit. Induction of APX activity due to MT is significantly inhibited by cPTIO [51]. These results indicate that NO may be involved in MT-induced stress tolerance by regulating antioxidant enzymes, thus resulting in reduced ROS accumulation.

Plants can defend against oxidative stress by regulating the regeneration pathway of non-enzymatic antioxidants including AsA, DHA, GSH, and GSSG. In the study, SNP or MT treatment significantly increases AsA and GSH content and significantly reduces DHA and GSSG content under Cd stress, but the effects are reversed by cPTIO under Cd conditions (Figure 6). MT increases antioxidant substance AsA and GSH accumulations in postharvest kiwifruit under low-temperature storage [66]. MT application also raises AsA levels in kiwifruit seedlings under heat stress [46]. NO increases the AsA and GSH content in tomato roots under copper stress [67]. We also found that the decrease of AsA/DHA and GSH/GSSG ratios caused by Cd stress is improved by SNP or MT treatment, but MT-induced increase of AsA/DHA ratio is inhibited by cPTIO under Cd stress conditions (Figure 7). Drought stress significantly reduces AsA/DHA and GSH/GSSG ratios and significantly increases NO content. Furthermore, jasmonic acid (JA) significantly enhances the above indicators, but these effects of JA are reversed by cPTIO, suggesting that NO is involved in JA-induced drought stress tolerance in wheat seedlings by the AsA-GSH cycle [68]. MT also significantly increases the decrease of GSH and total glutathione contents (GSH + GSSG) caused by salt stress, and MT content is modulated by NO, suggesting that the interaction between MT and NO plays an important role in response to abiotic stress [40]. These results suggest that NO may be involved in MT-induced stress tolerance by AsA-GSH cycle.

High levels of ASA/DHA and GSH/GSSG, which are mediated by DHAR, MDHAR, and GR, are considered to be important indices to estimate stress tolerance [69]. Cd treatment significantly inhibits MDHAR, MDHAR, and GR activities compared to control, whereas SNP or MT significantly reverses the inhibitive effect of Cd (Figure 9). cPTIO significantly inhibits the increase of MDHAR, MDHAR, and GR activities enhanced by MT under Cd stress (Figure 9). These results are in full agreement with the changes in *MDHAR*, *DHAR1*, *DHAR2*, and *GR* genes (Figure 9). During litchi cold storage, MT significantly enhances DHAR, MDHAR, and GR activities compared to control, but the increase due to MT is reversed by cPTIO [51]. Under salt stress, MT exhibits a significant increase in GR activity, the effect of MT on GR activity is inhibited by cPTIO in sunflower seedling cotyledons [40]. Thus, MT can mediate NO-induced salt tolerance in tomato seedlings via the regulation of AsA-GSH cycle-related enzymes and corresponding gene expression.

## 4. Materials and Methods

### 4.1. Plant Material and Growth Conditions

The ‘Micro-Tom’ (*Solanum lycopersicum* L.) tomato variety is used as the material in our experiments. First, we disinfect the seeds with 1% NaClO solution. Then the seeds are placed in a conical flask with pure water and put in an oscillating incubator (180 rpm, 28 °C) in the dark. After germination, we transfer them to opaque conical bottles with Hoagland solution and put the opaque conical bottles in the growth chamber (PRX-450C, Fuma Laboratory Instrument Co., Ltd., Shanghai, China) for 1 week. Then, seedlings of uniformly growing plants are collected and treated with different concentrations for 1 week: firstly, CdCl_2_ (chromium chloride, 50, 100, 200, and 300 μM); secondly, CdCl_2_ (200 μM) + MT (10, 50, 100, and 200 μM) and CdCl_2_ (200 μM) + SNP (sodium nitroprusside, as an NO donor, 10, 50, 100, and 200 μM); finally, CdCl_2_ (200 μM) + MT (100 μM), CdCl_2_ (200 μM) + SNP (100 μM), CdCl_2_ (200 μM) + SNP (100 μM) + MT (100 μM), and CdCl_2_ (200 μM) + MT (100 μM) + 10 μM cPTIO (2-4-carboxyphenyl-4,4,5,5-tetramethyl-imidazoline-1-oxyl-3-oxide, as an NO scavenger). The cPTIO concentration is obtained as described by Wei et al. [70]. All the treatments are added to the Hoagland solution, separately. Seedlings grown in Hoagland solution without any additional treatment are used as control. The experimental environment is maintained at 25 °C under 16 h light (250 µmol m^−2^ s^−1^ photons irradiance) and 8 h dark at 20 °C in 75% relative humidity. After the treatments, plant height and root length of seedlings are measured by vernier calipers. Then, all leaves in the different treatments are collected and divided into 0.1 g per sample for the study. When samples are used, they are collected randomly.

### 4.2. H_2_O_2_ Content

H_2_O_2_ content is determined according to Junglee et al. [71] with several modifications. The fresh leaf sample (0.1 g) is homogenized with liquid nitrogen and then transferred to 2 mL centrifuge tubes, which are stored in an ice bath. Then 1.5 mL 0.1% trichloroacetic acid (TCA) solution is added into the centrifuge tubes, which are centrifuged at 4 °C for 15 min at 12,000× *g*. After that, the supernatant (0.5 mL) is mixed thoroughly with 0.5 mL PBS (pH 7.0) solution and 1 mL of a 1 M KI solution. The mixture is kept at a constant temperature of 28 °C for 1 h. The absorbances are measured at 390 nm by ultraviolet spectrophotometer (UV-1800, Shimazu, Ibaraki, Japan) and the concentrations are calculated by using the standard curve of the H_2_O_2_ reference standard. In particular, the H_2_O_2_ standard curve concentrations are 0, 1, 2, 3, 4, and 5 mM, separately.

### 4.3. Malonaldehyde (MDA) Content

The measurement of MDA content is modified from the method described by Fazeli et al. [72]. Fresh leaf sample (0.3 g) is ground in an ice bath and 2 mL 0.05 M TCA solution is added. The homogenate is transferred to the centrifuge tube and centrifuged at 25 °C, 10,000× *g* for 5 min. Following this, 5 mL of 0.5% (*m*/*v*) thiobarbituric acid (TBA) solution is added to the supernatant. The extract is placed in a boiling water bath for 10 min, and then rapidly transferred to a cold-water bath. After cooling, the mixture is centrifuged at 25 °C, 10,000× *g* for 10 min again. The absorbances of the supernatant are determined at 532, 600, and 450 nm by ultraviolet spectrophotometer (UV-1800, Shimazu, Japan).

### 4.4. Ascorbic Acid (AsA) and Dehydroascorbic Acid (DHA) Content

The measurements of AsA and DHA contents are performed as previously reported by Arakawa et al. [73] with some modifications. The fresh leaf sample (0.5 g) is homogenized in an ice bath with 20 mL 50 g L^−1^ TCA, then 50 g L^−1^ TCA is applied to obtain the 100 mL solution. The homogenate is filtered, and the filtrate is the ascorbic acid extract. An extract of 1 mL is mixed with 0.5 mL 0.3 g L^−1^ FeCl_3_-alcohol, 1 mL absolute ethyl alcohol, 0.5 mL 0.4% (*m*/*v*) phosphoric acid-alcohol, 1 mL 50 g L^−1^ TCA solution, and 1 mL 5 g L^−1^ bathophenanthroline (BP)-alcohol. The mixture is held at a constant temperature of 30 °C for 60 min. Then, the absorbances are measured spectrophotometrically at 534 nm. Finally, we calculate AsA content using the standard curve of AsA reference standards, which are 0, 10, 20, 30, 40, 50, and 60 μg mL^−1^.

To measure the DHA content, 1 mL extract is mixed with 0.5 mL 60 mM dithiothreitol (DTT)-acetic acid. The value of pH is adjusted to 7–8 in the homogenate by adding the appropriate amount of NaHPO-NaOH drops. The reduction reaction of DHA is completed at room temperature after 10 min. Then, the mixture is added 0.5 mL 0.2 g mL^−1^ TCA. The subsequent steps are the same as for the AsA assay reaction system. The AsA standard curve is used to calculate the total ascorbic acid content. The DHA content is the subtraction of the total AsA content from the AsA content [74].

### 4.5. Reduced Glutathione (GSH) and Oxidized Glutathione (GSSG) Content

The content of GSH and GSSG are measured by GSH and GSSG content detection kits (Solarbio, Beijing, China) and ultraviolet spectrophotometer (UV-1800, Shimazu, Japan) following the manufacturer’s instructions, respectively.

### 4.6. Enzymatic Activities

The activities of the enzymes involved in the AsA-GSH cycle, including ascorbic acid oxidase (AAO, EC 1.10.3.3), ascorbate peroxidase (APX, EC 1.11.1.11), monodehydroascorbic acid (MDHAR, EC 1.6.5.4), dehydroascorbic acid reductase (DHAR, EC 1.8.5.1), and glutathione reductase (GR, EC 1.6.4.2), are determined using enzyme activity assay kits (Solarbio, China) and the ultraviolet spectrophotometer (UV-1800, Shimazu, Japan) according to the instructions of the manufacturer, separately.

### 4.7. RNA Isolation and Quantitative Real-Time Polymerase Chain Reaction (qRT-PCR)

We use TRIzol reagent to extract the total RNA. According to Huang et al. [75], we grind the tomato leaves (0.5 g) in liquid nitrogen as well as a pre-cooled mortar and then collect them in a centrifuge tube. TRIzol (1 mL) is added into the centrifuge tube and incubated at 4 °C for 10 min. Chloroform (200 μL) is added, incubated for 5 min, centrifuged at 4 °C and 12,000× *g* for 15 min, and the supernatant is collected. We add an equal volume of isopropanol into the supernatant and incubate it at −20 °C for more than 60 min. Next, with 75% ethanol, the supernatant is washed twice and collected into the adsorption column. Lastly, ddH_2_O without RNase-free is used to solubilize the RNA. We convert the extracted total RNA to single-stranded cDNA according to the recommendation of the manufacturer. We synthesize first-strand cDNAs, which are derived from differently treated RNA (500 ng) under 10 μL reaction conditions containing 2 μL AMV reverse transcriptase XL (AG, China) and 2.5 μM random primers. The reaction system contains: 6.8 mL RNase-free ddH_2_O, 0.6 mL 10 mM reverse primers, 10 mL 2 × SuperReal PreMix Plus, 2 mL cDNA, and 0.6 mL 10 mM forward primers.

RNA is reverse transcribed using the ABI Step One Plus system (Applied Biosystems, Carlsbad, CA, USA) and SYBR^®^ Premix Ex Taq ™ II (AG, China) according to the manufacturer’s instructions. PCR cycling conditions are as follows: 95 °C for 15 min and 40 cycles of 95 °C for 10 s and 60 °C for 20 s. We use the transcript level of *actin* as a normalization. The relative expression levels are indicated as the values at the specified time relative to the corresponding control samples. The primers used for PCR analysis are shown in Table 1.

### 4.8. Statistical Analysis

The experiment is conducted with three independent experiments (15 seedlings per replication) and the results are presented as mean values ± standard error (SE) [76]. The same and different letters indicate non-significant and significant differences in the figures, respectively [77]. The data analysis is carried out in Microsoft Excel 2021 (Microsoft Inc., Redmond, WA, USA). Analysis of variance (ANOVA) is performed using SPSS 22.0 (SPSS Institute Inc., Chicago, IL, USA) and treatment means are compared using the Tukey’s test at p < 0.05 level of probability [78]. All figures are prepared with OriginPro 2017 (OriginLab Institute Inc., Northampton, MA, USA).

## 5. Conclusions

In conclusion, Cd stress significantly inhibits the growth of tomato seedlings. The study reveals the important role that NO plays in melatonin in alleviating Cd stress in tomato seedlings. Moreover, the results indicate the mechanisms by which melatonin mediates NO to relieve Cd stress by improving the levels of antioxidant enzyme activities and their gene expression related to AsA-GSH cycle as well as antioxidant content and inhibiting oxidative damage of tomato seedlings. However, the mechanisms underlying melatonin and NO under abiotic stress are quite complex, and future research should be established to characterize the molecular mechanisms.

## Figures and Tables

**Table 1:** The primer sequence and accession number of genes.

Gene Symbol	Accession Number	Forward Primer	Reverse Primer
*AAO*	LOC101258916	5′-GAGCAATACGCACCTCAGATTCTCC-3′	5′-GCCAAGTTGAGTGAAGCCAATGC-3′
*AAOH*	LOC101263934	5′- GTGCTCTCATCCCTGTTCCTTTCG-3′	5′-CATCAGGTCTGCCAACGGTGTG-3′
*APX1*	LOC778224	5′-AGCAGTTTCCCACTCTCTCCCATG-3′	5′-CAACAGCAACAACACCAGCCAAC-3′
*APX6*	LOC778341	5′-GCTACCAGGTTCATTGCTCTTCTCC-3′	5′-TCGGCGAAGCGAATGTACTGAATC-3′
*MDHAR*	LOC778288	5′-CCATTTGGCGATTTCGGCTTGTAAG-3′	5′-ACACCCGCTCGCTCTCATCC-3′
*DHAR1*	LOC778229	5′-AAGAAGTGGAGTGTGCCTGAAAGC-3′	5′-CACGCATACAAGGACACGGTGAG-3′
*DHAR2*	LOC778343	5′-CACGAAGTTCAGAGCACCCAGAAG-3′	5′-CAGTCACCGAGCTTGTTAGGAGTTG-3′
*GR*	LOC100301931	5′-CCGCCCATTTATCCCAGACATTCC-3′	5′-GTCAGGCTTCGTTGGCAAATCAAG-3′
*Actin*	NC_015447	5′-AATGAACTTCGTGTGGCTCCAGAG-3′	5′-ATGGCAGGGGTGTTGAAGGTTTC-3′

## Data Availability

Not applicable.
